# University of Plymouth: Inaugural Lectures

**DOI:** 10.1155/S1463924694000179

**Published:** 1994

**Authors:** Les Ebdon

**Affiliations:** University of Plymouth UK


Journal of Automatic Chemistry, Vol. 16, No. 5 (September–October 1994), p. 151

### University of Plymouth: Inaugural Lectures

One evening in 1993, the University of Plymouth was host to a unique event. The Vice-Chancellor, Professor John Bull, was able to introduce inaugural lectures by the University's two newest Professors of Chemistry in a 'double feature' presentation. Professor Paul Worsfold joined the Plymouth Analytical Chemistry Research Unit in the Department of Environmental Sciences from the University of Hull; Professor Worsfold, who has a distinguished research record in the field of flow injection analysis, is Professor of Analytical Science. The other 'new' professor is a friend of the University of many years' standing and an internationally recognized leader of automated approaches to analytical chemistry, Professor Peter Stockwell, whom the University was delighted to honour with the title 'Visiting Professor'. Professor Stockwell will, of course, continue with his other responsibilities as editor of this journal and as Managing Director of PS Analytical Ltd, his company which specializes so successfully in laboratory automation. As Professor of Analytical Chemistry and Deputy Vice-Chancellor (Academic) at the University of Plymouth, it gives me particular pleasure to introduce the two papers presented that evening which are published in this edition of the *Journal of Automatic Chemistry*.

In composing this introduction I have been mindful that in little over 10 years the University of Plymouth (formerly Plymouth Polytechnic and Polytechnic South West) has established a reputation as one of Europe's leading centres of analytical chemistry. This has clearly been achieved by much hard work, but also by attracting top-quality analytical scientists to Plymouth and providing a climate in which they can flourish. To do this we have specialized in the liveliest areas of the subject, with a particular bias towards environmental problem-solving, process control and automated solutions. We have also been fortunate to have enjoyed the close co-operation of several analytical instrument manufacturers and other industrialists. One of our closest, and, certainly longest-standing, collaborations has been with PS Analytical. We have been proud to be a partner in several exciting projects which have led to the development of highly innovative and successful products, such as nebulizers, vapour generators and fluorescence detectors. We have particularly enjoyed the close co-operation of Professor Stockwell; this highly fruitful relationship has been a strength virtually throughout the whole period of existence of both the analytical chemistry research unit at Plymouth and of PS Analytical.

I hope that readers will agree that these two articles demonstrate the mastery of two worthy professors. Professor Worsfold demonstrates that flow injection can bring a new dimension to environmental monitoring that allows, for example, remote and selective monitoring of water quality in natural and polluted systems. Professor Stockwell gives an overview of automatic analysis that only an acknowledged world expert can give; in particular it sets the challenge to the analytical chemist as to the new skills that must be acquired to benefit fully from the opportunities that computers have brought to the laboratory. I hope, too, that these papers illustrate that there is a synergy between academia and industry and that the University of Plymouth is right to believe that not only its students, research and reputation will benefit from closer collaboration but that society at large has much to gain from such co-operation.

**Les Ebdon**
*University of Plymouth*

0142-0453/94 $10.00 © 1994 Taylor & Francis Ltd.

